# Evaluating histone modification analysis of individual preimplantation embryos

**DOI:** 10.1186/s12864-024-09984-8

**Published:** 2024-01-18

**Authors:** Yiren Zeng, Yoichiro Hoshino, Kazuki Susami, Shinnosuke Honda, Naojiro Minami, Shuntaro Ikeda

**Affiliations:** Laboratory of Reproductive Biology, Graduate School of Agriculture, Kyoto University, Kyoto, 606-8502 Japan

**Keywords:** Preimplantation embryo, Histone, Epigenetic diagnosis

## Abstract

**Background:**

We previously reported a modification of the CUT&Tag method (NTU-CAT) that allows genome-wide histone modification analysis in individual preimplantation embryos. In the present study, NTU-CAT was further simplified by taking advantage of the Well-of-the-Well (WOW) system, which enables the processing of multiple embryos in a shorter time with less reagent and cell loss during the procedure (WOW-CUT&Tag, WOW-CAT).

**Results:**

WOW-CAT allowed histone modification profiling from not only a single blastocyst but also from a portion of it. WOW-CAT generated similar H3K4me3 profiles as NTU-CAT, but they were closer to the profiles produced by chromatin immunoprecipitation-sequencing, such as a valley-like trend and relatively lower false positive rates, indicating that WOW-CAT may attenuate the bias of Tn5 transposase to cut open chromatin regions. Simultaneous WOW-CAT of two halves of single blastocysts was conducted to analyze two different histone modifications (H3K4me3 and H3K27ac) within the same embryo. Furthermore, trophectoderm cells were biopsied and subjected to WOW-CAT in anticipation of preimplantation diagnosis of histone modifications. WOW-CAT allowed the monitoring of epigenetic modifications in the main body of the embryo. For example, analysis of H3K4me3 modifications of *XIST* and *DDX3Y* in trophectoderm biopsies could be used to sex embryos in combination with quantitative PCR, but without the need for deep sequencing.

**Conclusions:**

These results suggest the applicability of WOW-CAT for flexible epigenetic analysis of individual embryos in preimplantation epigenetic diagnosis.

**Supplementary Information:**

The online version contains supplementary material available at 10.1186/s12864-024-09984-8.

## Background

Genome research has been revolutionized by the availability of massively parallel sequencing and its dramatically reduced costs, so the current difficulties are mostly related to the development of methods to obtain high-quality chromatin fragments for sequencing [1]. Epigenomic profiling of preimplantation embryos may be useful for locating abnormal epigenetic modifications and revealing the reason for compromised embryo quality caused by adverse environmental effects that vary according to the conditions and protocols for embryo production such as ovarian stimulation and in vitro production [2–6]. Temporally and spatially appropriate epigenetic modifications play a crucial role in gene expression in preimplantation embryos [7].

As the last stage before embryo transfer, the quality of blastocysts produced in vitro requires close attention, and the methods for detecting epigenetic modifications of individual embryos are continually being improved. Genome-wide investigations of post-translational histone modifications in early embryos used to be performed using mainly chromatin immunoprecipitation-sequencing (ChIP-seq) [8–10]. However, because ChIP-seq requires a large number of cells, it is difficult to use this approach to analyze a single embryo that usually contains ~ 100 cells. To overcome the disadvantages of ChIP-seq for analyzing single or small numbers of preimplantation embryos, several methods have been developed such as enzyme-tethering methods for unfixed cells, in which a specific protein of interest is targeted in situ (within the cells) and then profiled genome-wide. For example, Cleavage Under Targets and Release Using Nuclease (CUT&RUN), which is based on Laemmli’s Chromatin ImmunoCleavage strategy [11], targets a chromatin protein through successive binding of a specific antibody and a protein A (pA)-micrococcal nuclease fusion protein that cuts and releases the nearby DNA fragments into the reaction supernatant for subsequent adaptor tagmentation [12, 13]. Then, Cleavage Under Targets and Tagmentation (CUT&Tag) was developed, in which a fusion protein of Tn5 transposase and pA (pA-Tn5) is used instead of pA-micrococcal nuclease, enabling the cutting and adaptor tagmentation of the fragments in situ simultaneously [14, 15]. CUT&Tag was conceptualized as a more efficient alternative to ChIP-seq and the CUT&RUN method, successfully generating H3K27me3 profiles from as few as 60 human K562 cells [15]. Although CUT&Tag profiles have been obtained from > 1000 bovine blastomeres in a work by Zhou et al. [16], investigations on mouse blastomeres indicate that postponing the binding step using concanavalin-coated magnetic beads after antibody incubation can safeguard delicate blastomeres, yielding CUT&RUN profiles from as few as 100 blastomeres [17, 18].

Nowadays, CUT&Tag is used to profile representative histone modifications in single blastocysts without binding to a solid phase (NON-TiE-UP CUT&Tag [NTU-CAT]), which has made this method more readily applicable [19]. After the successful implementation of NTU-CAT, we hypothesized that this method could be simplified and optimized by using the Well-of-the-Well (WOW) system [20, 21], which was named WOW-CUT&Tag (WOW-CAT). With this method, a maximum of 13 embryos could be treated simultaneously in one WOW dish until the tagmentation step by only liquid exchange without touching the embryos, which saved a considerable amount of time and reaction reagents compared with NTU-CAT. The reduction of systematic errors and cell loss with this approach was considered to facilitate the recovery of DNA that could be sequenced and to enable the profiling of histone modifications using not only a single blastocyst but also a small part of it.

## Results

### Schema for WOW-CAT

**Fig. 1:**
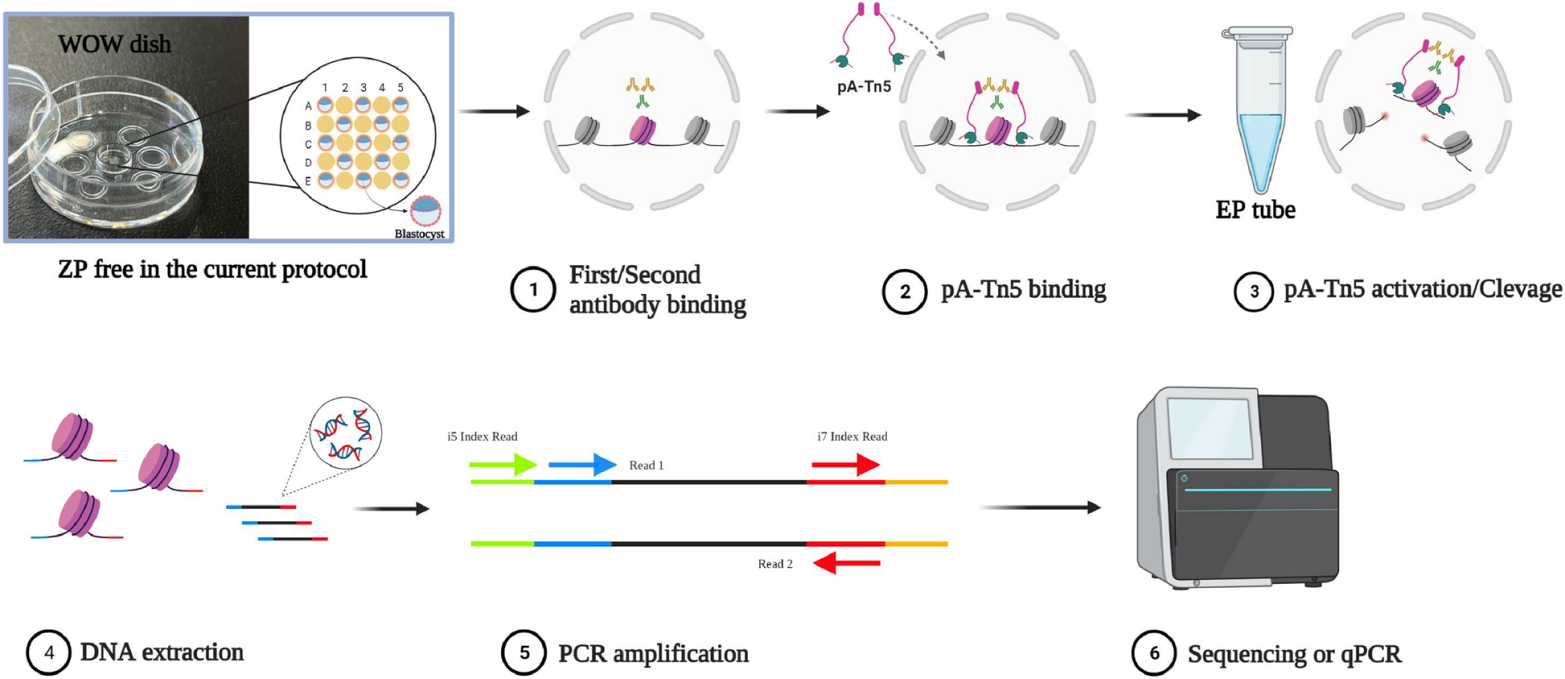
Schematic diagram of WOW-CAT. See the “Methods” section for details. All icons except the WOW dish picture are from Biorender.com

The schema for WOW-CAT is shown in Fig. 1. After removal of the zona pellucida or biopsy, the individual embryos or cell masses were transferred into separate microwells in the WOW dish containing a primary antibody solution with a detergent (digitonin) and incubated. Although we had 25 microwells per dish, the maximum number of embryos processed per dish was 13 because the wells were used so that samples were not adjacent to each other vertically and horizontally. Subsequent secondary antibody reactions and the tethering of pA-Tn5 fusion protein with sequencing adapters were performed by exchanging the respective reaction solutions in the WOW dish. The tagmentation reaction with pA-Tn5 activation was performed in microtubes and the tagmented DNA was extracted and PCR amplified using index primers, and the library after purification was used for sequencing.

### H3K4me3 profile of whole blastocysts assessed by WOW-CAT

**Fig. 2:**
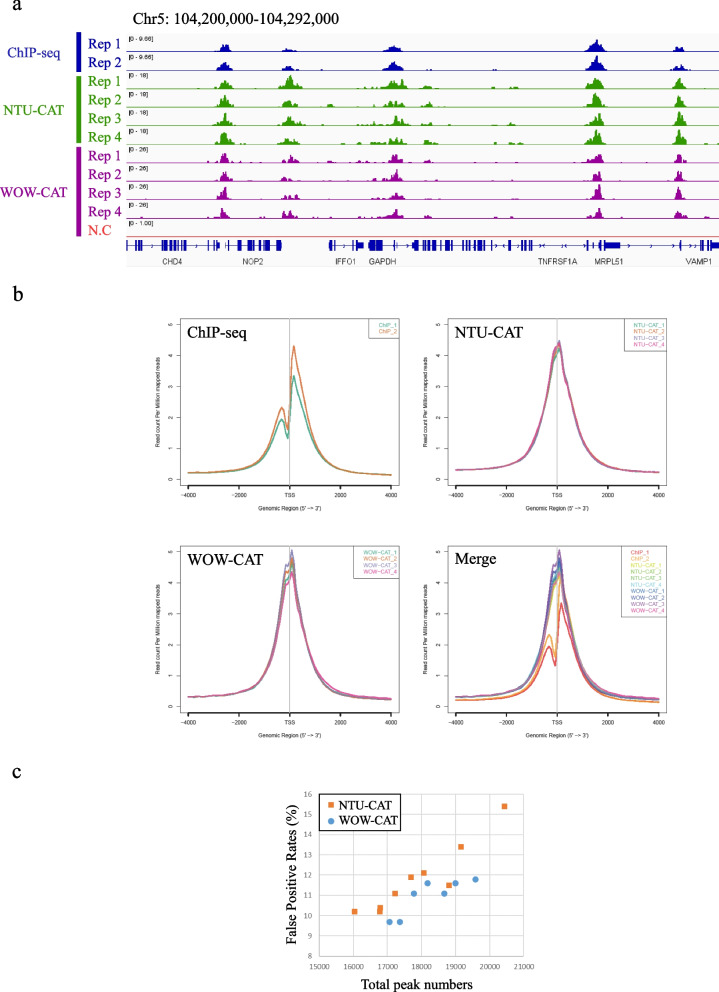
Comparison of WOW-CAT, NTU-CAT, and ChIP-seq results for H3K4me3 in bovine blastocysts. **a** A snapshot of the WOW-CAT peaks from four replicates, alongside the peaks from our previous ChIP-seq and NTU-CAT analyses of blastocysts for the H3K4me3 modification. **b** The average profile plots of H3K4me3 signals around the TSSs in these experiments. **c** Scatter plots of total peak counts and false positive rates (FPRs) for WOW-CAT (present study) and NTU-CAT [19]

Since we had previously validated NTU-CAT and obtained comparable results to ChIP-seq for the H3K4me3 modification [19], we initially targeted this modification for WOW-CAT application. A snapshot of the WOW-CAT peaks for the H3K4me3 modification from four replicates (i.e., four single blastocysts) is shown in Fig. 2a, alongside the peaks from our previous ChIP-seq [22] and NTU-CAT [19] analyses of blastocysts. The overall landscape depicted by the location and shape of the peaks was very similar among the three methods, except for subtle differences in the shapes of the peaks (Fig. 2a). Figure 2b shows the average profile plots of the H3K4me3 signal around the transcription start sites (TSSs) in these experiments. A striking difference between the NTU-CAT and ChIP-seq profiles was that the valley-like shapes near TSSs detected in ChIP-seq were not detected in NTU-CAT, which was in line with the findings of other studies on CUT&Tag [15, 23]. In contrast, WOW-CAT captured a little more of the valley-like shapes, although not as much as ChIP-seq. We calculated the false positive rate (FPR), which could be caused by the bias of Tn5 transposase toward open chromatin. The FPR was determined as the number of peaks that did not overlap with ChIP-seq, but did overlap with the ATAC-seq peaks, divided by the total number of peaks, as proposed by Wang et al. [24]. Figure 2c shows scatter plots of total peak counts and FPRs for WOW-CAT and NTU-CAT. WOW-CAT consistently exhibited a lower FPR per similar number of peaks than NTU-CAT. Pairwise comparisons of H3K4me3 signals showed a high correlation among the methods and replicates (Fig. S1).

### Simultaneous H3K4me3 and H3K27ac profiling within single blastocysts assessed by WOW-CAT

**Fig. 3:**
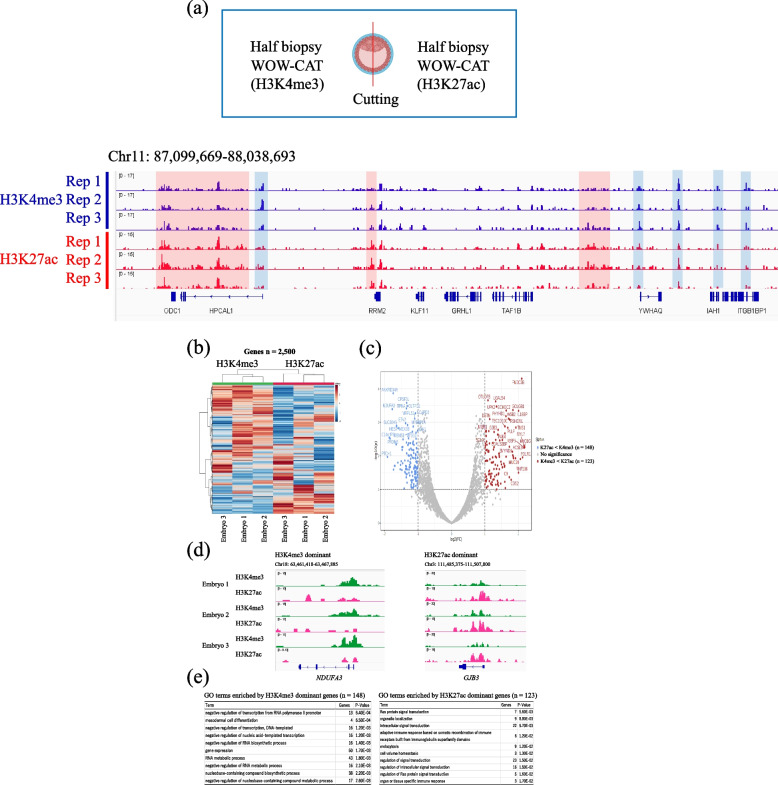
Simultaneous profiling of H3K4me3 and H3K27ac within the same blastocysts assessed by WOW-CAT. **a** The results were similar to those reported by Zhou et al. [16] in terms of the common (unshaded), H3K4me3-dominant (blue-shaded), and H3K27ac-dominant (red-shaded) areas, respectively. Blastocyst icon is from Biorender.com. **b**,** c** Heatmap and volcano plot, respectively, of the normalized H3K4me3 and H3K27ac modifications within three single blastocysts. **d** Examples of H3K4me3- and H3K27ac-dominant genes. **e** Gene Ontology terms enriched by H3K4me3- and H3K27ac-dominant genes

WOW-CAT allows the reaction solution to be changed without moving the cells while they remain at the bottom of the dish, making it easier to complete the immuno-tethering process without losing the samples, even with smaller cell masses. Therefore, we performed the simultaneous profiling of two different histone modifications within the identical single blastocysts, which has never been reported. To accomplish simultaneous profiling, a whole blastocyst was cut equally into two parts, so that each half contained the ICM and TE (Fig. 3a). Then, each half was subjected to WOW-CAT to profile the H3K4me3 and H3K27ac modifications, respectively. Figure 3a shows the same region as reported by Zhou et al. [16], who used 1000–1500 blastomeres for CUT&Tag for these modifications. Our present results were similar to those of Zhou et al. in terms of the common (unshaded), H3K4me3-dominant (blue-shaded), and H3K27ac-dominant (red-shaded) areas. The average profile plots of the H3K4me3/H3K27ac signals around the TSSs and gene bodies are shown in Fig. S2. Highly similar but different modification profiles between H3K4me3 and H3K27ac around the TSSs were detected in this experiment. Figure 3b and c show the heatmap and volcano plot, respectively, of the normalized H3K4me3 and H3K27ac modifications within three single embryos. These analyses clearly demonstrated H3K4me3- or H3K27ac-dominant genes even within the same embryo (e.g., *NDUFA3* and *GJB3*, respectively) (Fig. 3d). The genes showed enrichment in the biological processes exclusively related to gene expression and signal transduction, respectively (Fig. 3e).

### H3K4me3 profile of biopsied single blastocysts assessed by WOW-CAT

**Fig. 4:**
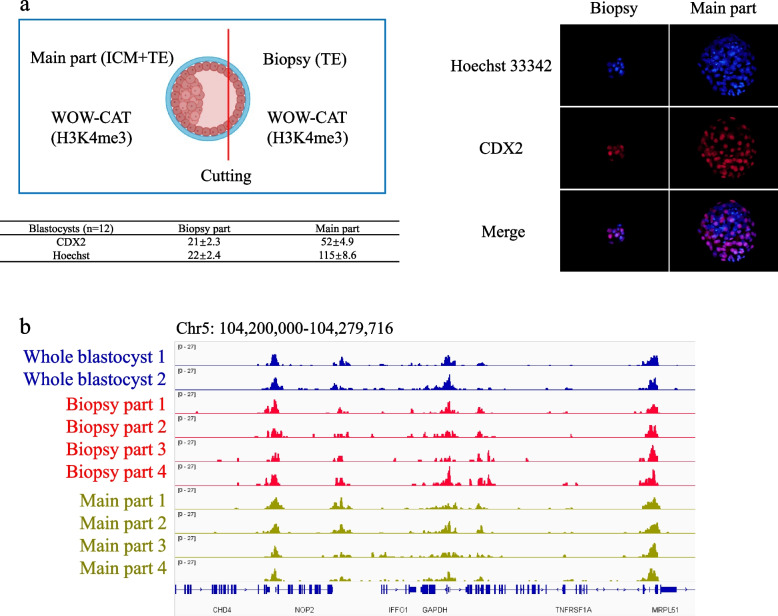
H3K4me3 profile of biopsied single blastocysts assessed by WOW-CAT. **a** The precision of TE cell biopsy examined by immunofluorescent staining of *CDX2* as a TE cell-specific marker in combination with Hoechst 33342 staining of total cell nuclei. The table shows the cell numbers of each part (mean ± standard error of the mean). Blastocyst icon is from Biorender.com. **b** A snapshot of the WOW-CAT H3K4me3 peaks of the whole, biopsied, and remaining (main) parts of blastocysts

To preserve the integrity of blastocysts for future embryo transfer, we biopsied only as few TE cells as possible to perform WOW-CAT. The precision of the TE cell biopsy was examined by immunofluorescent staining of CDX2 as a TE cell-specific marker in combination with Hoechst 33342 staining of total cell nuclei (Fig. 4a). The high rate of average CDX2/Hoechst-stained cells (21 in 22, 95%) in the biopsied part indicated that the TE cells were precisely excised. In addition, the absence of H3K4me3 modifications of *NANOG* (an ICM-specific marker) in the biopsied part further showed the precision of the TE cell biopsy (Fig. S3). A snapshot of the WOW-CAT H3K4me3 peaks of the whole, biopsied, and remaining (main) parts of the blastocysts is shown in Fig. 4b. The overall landscape depicted by the location and shape of the peaks was similar among the different samples.

### Sex identification: preliminary application of WOW-CAT for embryonic diagnosis

**Fig. 5:**
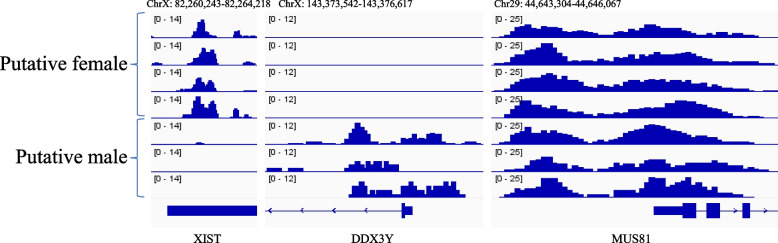
H3K4me3 modifications at *XIST* and *DDX3Y* as female- and male-specific markers, respectively, from NGS-based WOW-CAT. *MUS81* is an internal control that shows stable sex independent H3K4me3 modifications

With the successful construction of DNA libraries for the enrichment of specific histone modifications from biopsied TE cells by WOW-CAT, we developed a qPCR-based protocol to detect important histone markers from these libraries, which could directly reflect the properties of the remaining part of the blastocyst. We selected H3K4me3 modifications at *XIST* and *DDX3Y* as female- and male-specific sex identification markers, respectively, from the results of NGS-based WOW-CAT (Fig. 5). Figure 5 shows that most embryos had only one modification, either *XIST* or *DDX3Y*, suggesting they were female and male embryos, respectively. We designed primers for these genes at areas with abundant H3K4me3 modifications.

**Fig. 6:**
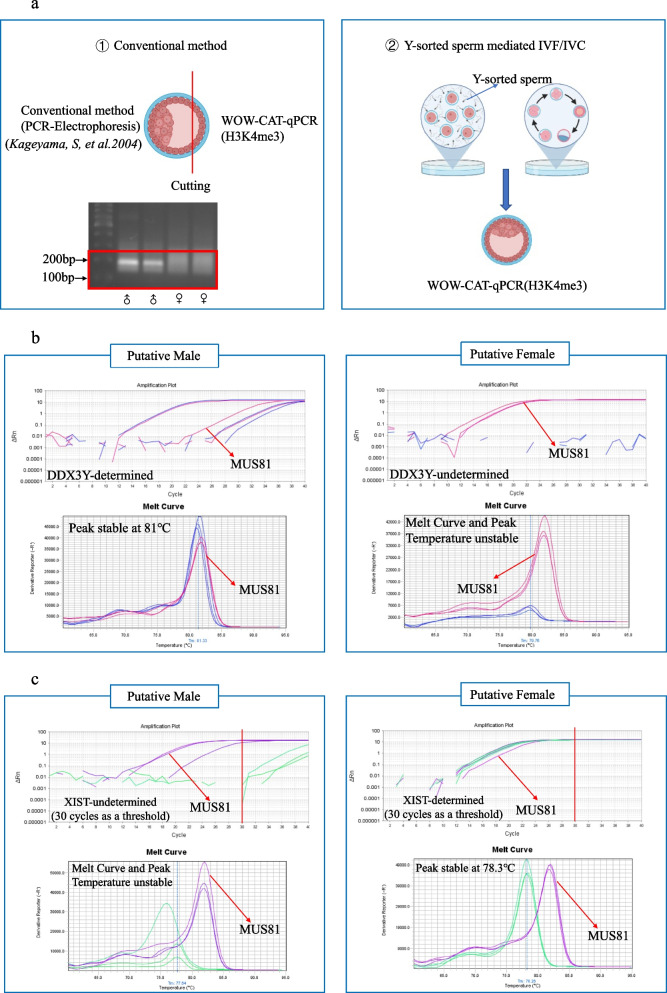
Sex identification accuracy validation. **a** Schema of comparison between ① the conventional sex identification method and ② IVF using Y chromosome-sorted sperm and WOW-CAT-qPCR-based sex identification. All icons except the electrophoresis picture are from Biorender.com. **b** Comparison of the results between the conventional sex identification method and WOW-CAT-qPCR for *DDX3Y*. **c** Comparison between the conventional sex identification method and WOW-AT-qPCR for *XIST*. For the amplification plot, threshold for the rise of the curve was set to 30 cycles

We first checked how well the results from the conventional sex identification method [25] matched those from histone modification-based sex identification using WOW-CAT and subsequent qPCR with the 18 embryos remaining after TE cell biopsy (Fig. 6a). The conventional sex identification method [25] determined 7 as male and 11 as female. In qPCR, *MUS81* was selected as an internal control to indicate experimental stability when it showed a positive amplification curve and stable melt curve peak temperature at approximately ~81 °C. *DDX3Y* was positively determined by the appearance of an amplification curve and stable melt curve with a peak temperature of approximately ~81 °C, while it was considered undetermined in the absence of an amplification plot or presence of an unstable melt curve. Taking these criteria, 6 out of 7 (86%) male and all 11 (100%) female embryos were identified as such also in WOW-CAT-qPCR for *DDX3Y* (total matching was 17/18 = 94.4%) (Fig. 6b). For *XIST*, setting the threshold for the rise of the amplification curve to 30 cycles with a melt curve peaking at ~78 °C resulted in 100% concordance (17/17, one male embryo was used in another experiment and was excluded from the calculation) (Fig. 6c).

**Fig. 7:**
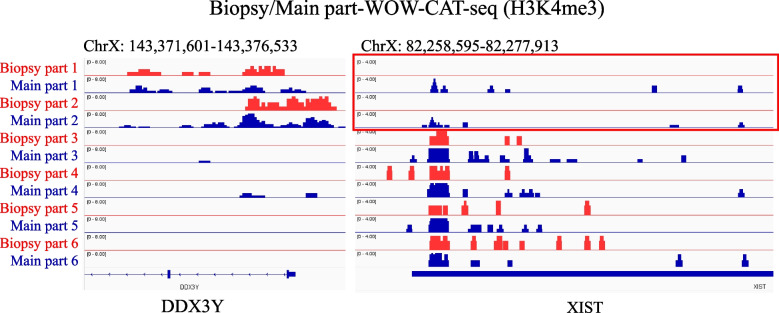
NGS-based WOW-CAT results at *XIST* and *DDX3Y* in the biopsied and main parts of blastocysts

In addition, we assessed the embryos derived from IVF using Y chromosome-sorted sperm. WOW-CAT-qPCR identified 10 embryos out of 12 (83.3%) as male using *DDX3Y* as a marker, consistent with the approximately 90% sex ratio of Y chromosome-sorted sperm. In contrast, *XIST* sometimes showed unexpected amplification from presumptive male (*DDX3Y*+) samples, which was consistent with the results of the NGS data (H3K4me3 modifications in both genes) in NTU-CAT (Fig. S4). Thus, the *XIST* marker did not seem to be reliable for screening whole embryo-derived WOW-CAT DNA libraries. We assessed the biopsied/main parts of the NGS-based WOW-CAT results again and found that the biopsied (TE) parts showed nearly no modification of *XIST* in putative male (*DDX3Y*+) embryos while their main parts unexpectedly harbored *XIST* modifications (Fig. 7). Therefore, the Y chromosome-sorted sperm-derived embryos (using whole embryos) may not be suitable to verify the efficacy of WOW-CAT-qPCR using *XIST* as a marker because some male embryos showed modifications of not only *DDX3Y* but also *XIST*, particularly in the ICM-containing part of the embryo. As assumed, there was no correlation between *XIST* marker-based WOW-CAT-qPCR sex identification and expected sex (male) derived from Y chromosome-sorted sperm (data not shown).

## Discussion

The conventional CUT&Tag method [14, 15] can analyze histone modifications in a small number of cells. This approach utilizes concanavalin A-coated magnetic beads to fix dispersed cells to the solid phase in order to facilitate handling and processing of the cells. However, preimplantation embryos are cell masses that can be individually transferred to any reaction solution in the experimental process using a fine pipette. Recently, the new method of NTU-CAT [19] was developed that can handle embryos with good permeability of antibodies even without a solid phase. With the successful implementation of NTU-CAT, we further modified it by taking advantage of the WOW system [20, 21], which enables the processing of multiple embryos in a shorter time with less reagent. The superiority of WOW-CAT over NTU-CAT in terms of typical reagents and the number of well transferring times is listed in the Supplementary Table 1. Because WOW-CAT required only liquid exchange without well transfer until the tagmentation step, systematic errors and cell loss were reduced and facilitated the profiling of histone modifications using not only a single blastocyst but also a small part of it.

As a result, WOW-CAT generated genome-wide profiles of representative H3K4me3 histone modifications from single embryos, which were comparable to the results obtained using the conventional ChIP-seq method and NTU-CAT. We demonstrated the overall similarity of the signals detected among these three methods (Fig. 2a, b, and Fig. S1). The shape of the average profile of the peaks near the TSSs of genes differed among the three methods, such that the “valleys” detected by ChIP-seq were not observed with NTU-CAT but were slightly detected with WOW-CAT (Fig. 2b). The difficulty in detecting “valleys” is a general feature of CUT&Tag experiments [15, 23] and may be due to the bias of Tn5 transposase to preferentially cut open chromatin regions [24]. The magnitude of this bias is estimated to generate 10–15% false positive peaks per sample, which are detected possibly due to the open chromatin bias of Tn5 transposase [19]. The valley-like trend of WOW-CAT, which indicates an intermediate form between ChIP-seq and NTU-CAT, suggests that WOW-CAT may alleviate this bias of Tn5 transposase. Encouragingly, the alleviation of Tn-5 transposase bias in WOW-CAT was further demonstrated by a consistently lower FPR per similar number of peaks than NTU-CAT (Fig. 2c). In addition, high correlations of the signals per genomic bin were obtained between WOW-CAT and ChIP-seq (Pearson correlation = 0.88–0.91), which showed nearly no difference between NTU-CAT and ChIP-seq (Pearson correlation = 0.89–0.91), between WOW-CAT and NTU-CAT (Pearson correlation = 0.95–0.97), and within the WOW-CAT replicates (Pearson correlation = 0.96–0.97, Fig. S1).

The reduction of systematic errors by WOW-CAT compared with NTU-CAT made it possible to obtain results from a cell mass smaller than a whole blastocyst. Then, simultaneous WOW-CAT of two equal parts of the embryo was conducted for H3K4me3 and H3K27ac, respectively. Figure 3a shows the same region as reported by Zhou et al. [16], who used 1000–1500 blastomeres for CUT&Tag to detect these modifications. A high similarity was demonstrated in the common (unshaded), H3K4me3-dominant (blue-shaded), and H3K27ac-dominant (red-shaded) areas, indicating a high similarity between conventional CUT&Tag using a group of embryos and WOW-CAT using even a part of single embryos. The average profile plots of the H3K4me3/H3K27ac signals around the TSSs are shown in Fig. S2. The highly similar valley-like trend might be due to the same gene expression-promoting function of H3K4me3 and H3K27ac. The H3K4me3 signals around the TSSs were higher than those of H3K27ac, which is also consistent with the results shown by Zhou et al., who used the same antibodies [16]. This is the first simultaneous detection of H3K4me3 and H3K27ac modifications within identical single blastocysts. Figure 3b and c show the heatmap and volcano plot, respectively, of the normalized H3K4me3 and H3K27ac modifications within three single embryos. Among the 2500 genes analyzed, 148 and 123 genes were H3K4me3- and H3K27ac-dominant, respectively, and the other genes showed a balanced state. Intriguingly, the H3K4me3- and H3K27ac-dominant genes are involved in different biological processes. For example, the protein encoded by the H3K4me3-dominant gene *NDUFA3* is a subunit of ubiquinone, which is located in the mitochondrial inner membrane and is the largest of the five members of the electron transport chain [26]. The protein encoded by the H3K27ac-dominant gene *GJB3* is a component of gap junctions, which provide a route for the cell-to-cell diffusion of low molecular weight materials [27]. Although H3K4me3 and H3K27ac are both gene expression-promoting modifications, this is the first time that subtle differences in their dominance in different genes and biological processes at the level of an identical blastocyst have been shown (Fig. 3c–e).

We next attempted to biopsy a small part of a single blastocyst for WOW-CAT to establish a method for diagnosing the properties of embryos. We biopsied only TE cells to preserve the integrity of the blastocysts for future embryo transfer. The precision of TE cell biopsy was examined (Fig. 4a) and the high rate of average CDX2/Hoechst-stained cells (21 out of 22, 95%) in the biopsied part indicated that the TE cells were sampled precisely. In addition, the absence of H3K4me3 modifications of *NANOG* (an ICM-specific marker) in the biopsied part further showed the precision of TE cell biopsy (Fig. S3). A snapshot of the WOW-CAT H3K4me3 peaks of the whole, biopsied, and remaining (main) parts of blastocysts is shown in Fig. 4b. The overall landscape depicted by the location and shape of the peaks was almost the same among them. This indicated that the H3K4me3 modification level of TE cells can generally reflect that of the remaining part of the blastocyst, suggesting the possible usage of TE (biopsy) cell modifications for diagnosis. With the successful construction of DNA libraries for the enrichment of specific histone modifications from biopsied TE cells by WOW-CAT, we developed a qPCR-based detection protocol for important histone markers from TE cell-derived DNA libraries, which directly reflected the properties of the remaining part of the blastocyst.

We selected H3K4me3 modifications at *XIST* and *DDX3Y* as possible sex-identification markers for females and males, respectively, from the NGS-based WOW-CAT analysis (Fig. 5), in which embryos generally showed only one modification, either *XIST* (activated only in females) or *DDX3Y* (a Y chromosome gene), suggesting they were female and male embryos, respectively.

Then, we validated the accuracy of the WOW-CAT-qPCR method for sex identification by comparing it with the conventional sex identification method [25] and by using Y chromosome-sorted sperm-derived embryos. The results for *XIST* and *DDX3Y* were well matched with those of the conventional sex identification method, but only *DDX3Y* matched well in the Y chromosome-sorted sperm-based method (Fig. 6a–c). This may be explained by the fact that we only used the biopsied (TE) part in the comparison with the conventional sex identification method, which was applied to the remaining main part of the same embryos in the present study, while we used whole blastocysts in the comparison with the Y chromosome-sorted sperm-based experiment. Thus, we checked the NGS results of WOW-CAT for the biopsied and main parts again and found that the main part containing the ICM had a faint *XIST* modification, despite the presence of the *DDX3Y* modification, while the TE part was completely free of the *XIST* modification within the same embryos (Fig. 7). Therefore, the comparison with the conventional sex identification method was considered to verify the accuracy of WOW-CAT-qPCR, but the Y chromosome-sorted sperm-derived embryos used as whole embryos may not be suitable for this purpose. In addition, this diagnostic method was designed to use the TE part to preserve the integrity of blastocysts for future embryo transfer. Thus, it does not matter that *XIST* sometimes shows unexpected amplification in presumptive male (*DDX3Y*+) samples (Fig. S4). However, the reason for the inconsistency of the modification level between the TE part and the remaining part in male blastocysts is unclear and more research is required to explain this interesting phenomenon.

As we successfully biopsied the TE cells for WOW-CAT and obtained *XIST/DDX3Y* amplification from qPCR, we consider that a basic diagnostic method for embryos was established, at least in terms of sex identification. It is anticipated that the identification of useful epigenetic modifications will continue to progress for histone modifications, which will allow for the evaluation of the quality of embryos beyond sex [6]. If useful markers are identified, they may be used for the quality control of embryos themselves and embryo production protocols contributing to improved embryo quality in assisted reproductive technologies (ART).

We used a bovine model because bovine embryos are more similar to human embryos than rodent models in many respects, including mono-ovulatory nature, gamete size, embryonic developmental speed, blastocyst cell numbers, and the timing of embryonic genome activation, which makes it a clinically important model for the study of human embryos [28]. However, further validation in other experimental animal models is needed to explore a wider range of applications for WOW-CAT.

## Conclusion

WOW-CAT enables the profiling of genome-wide histone modifications from not just a single blastocyst but also from a portion of it. By using this method, histone modifications were analyzed in two halves of the same single blastocyst and in the TE cell part for the first time. By combining WOW-CAT with qPCR for the TE cell part, information on specific histone markers can be detected, which reflects the properties of the remaining part of the embryo without the need for deep sequencing. These results suggest the applicability of WOW-CAT for flexible epigenetic analysis in individual embryos as well as for preimplantation epigenetic diagnosis. With the anticipation of discovering markers indicative of embryo quality, this procedure can be further perfected to ensure embryo quality control before transfer, which will be significant for improving the efficacy of ART.

## Methods

### In vitro production of bovine blastocysts

This study was approved by the Animal Research Committee of Kyoto University (permit numbers R3–10, R4–10, and R5–10) and was conducted in accordance with the Regulations on Animal Experimentation at Kyoto University. The bovine ovaries used in this study were purchased from a commercial abattoir as by-products of meat processing, and the frozen bull semen (conventional and Y chromosome-sorted) used for in vitro fertilization (IVF) was also commercially available. In vitro production of bovine embryos by IVF was performed as previously described [22, 29]. Blastocyst-stage embryos at 168–192 h post-IVF (days 7–8) were collected individually.

### Blastocyst biopsy

Blastocyst biopsy was performed as described by de Sousa et al. [30] with minor modifications. Briefly, blastocysts were cut using a micromanipulator (Leica Microsystems, Wetzlar, Germany) and a stainless-steel blade at an angle of 30° (Bio-Cut-Blades Feather; Feather Safety Razor Co., Osaka, Japan). Embryos were micromanipulated on a 90 × 15 mm Petri dish (AS ONE Corporation, Osaka, Japan) containing 200 μL holding medium consisting of TCM–199 and Hanks’ salts (Invitrogen, Carlsbad, CA) supplemented with 0.3% (w/v) bovine serum albumin (BSA; Sigma-Aldrich, St. Louis, MO).

### WOW-CAT

For whole blastocysts, the zona pellucida was freed from the embryos by 0.5% (w/v) pronase treatment and washed with phosphate-buffered saline (PBS) containing 0.01% (w/v) polyvinyl alcohol (PVA) and 1% (v/v) Protease Inhibitor Cocktail (PIC) (PBS-PVA-PIC) before allocation into the WOW dish (LinKID micro25 Culture Dish; Dai Nippon Printing, Tokyo, Japan). The biopsies and remaining parts of the blastocysts were washed with PBS-PVA-PIC before placing in the WOW dish. Then, individual samples were transferred into separate microwells in the WOW dish by individual pipettes to avoid cross-contamination and all the treatments onward were conducted in one WOW dish until pA-Tn5 binding by only liquid exchange. The basal kit for CUT&Tag was a CUT&Tag-IT Assay Kit (Active Motif, Carlsbad, CA). Dig-Wash buffer and Dig-300 buffer in the kit were supplemented with 0.01% (w/v) PVA to avoid cell adhesion to the wall of the dishes or pipettes. Primary antibody binding was performed in the WOW dish with 100 μL Antibody Buffer, which contained 2 μL (2.8 and 5.6 μg for H3K4me3 and H3K27ac, respectively) of the primary antibodies (C15410003 and C15410196 for H3K4me3 and H3K27ac, respectively; Diagenode, Denville, NJ), 0.05% (w/v) digitonin, and 1% (v/v) PIC. The blastocysts were incubated at 4 °C overnight with gentle shaking (400 rpm). A negative control was set by omitting the primary antibodies. After the primary antibody reaction, the primary antibody buffer was replaced with 100 μL Dig-Wash buffer containing a secondary antibody (guinea pig anti-rabbit IgG antibody, 1 μL), 0.05% (w/v) digitonin, and 1% (v/v) PIC and incubated for 1 h at room temperature (400 rpm). After three washes with 100 μL Dig-Wash Buffer supplemented with digitonin and PIC, pA-Tn5 binding was performed using 100 μL Dig-300 Buffer replacement, which contained 1 μL pA-Tn5 transposomes, 0.01% (w/v) digitonin, and 1% (v/v) PIC and incubated for 1 h at room temperature (400 rpm). After three washes with 100 μL Dig-300 Buffer supplemented with digitonin and PIC, the cells were transferred to individual microcentrifuge tubes (Eppendorf 0030 108.051) containing 125 μL Tagmentation Buffer with 0.01% (w/v) digitonin and 1% (v/v) PIC and incubated for 1 h at 37 °C without shaking.

After tagmentation, 4.2 μL of 0.5 M EDTA, 1.25 μL of 10% SDS, and 1.1 μL of 10 mg/mL proteinase K were added to each tube and incubated for 1 h at 55 °C with vigorous shaking (1300 rpm). After cooling to room temperature, SPRIselect beads (145 μL; Beckman Coulter, Brea, CA) were added to each tube, vortexed for 1 min, and allowed to incubate for 10 min at room temperature. The tubes were placed on a magnetic stand for 4 min to collect the magnetic beads and the liquid was removed. The beads were washed twice with 1 mL of 80% ethanol. After drying the bead pellets for 2–5 min, 35 μL DNA Purification Elution Buffer was added and the tubes were vortexed and left to stand for 5 min at room temperature. The tubes were placed on a magnetic stand for 4 min to collect the magnetic beads and the liquid containing tagmented DNA was transferred to PCR tubes.

PCR amplification of sequencing libraries was performed in a volume of 50 μL using 30 μL tagmented DNA and i7 and i5 indexing primers according to the manufacturer’s protocol. The PCR conditions were as follows: 72 °C for 5 min; 98 °C for 30 s; 20 cycles for blastocysts (23 cycles for biopsies and 21 cycles for the remaining parts or halved blastocysts) of 98 °C for 10 s and 63 °C for 10 s; final extension at 72 °C for 1 min; and hold at 10 °C. Post-PCR library purification was performed with 55 μL SPRIselect beads (vortex for 1 min, stand for 5 min, and bead collection for 4 min) and 180 μL of 80% ethanol as described above. Finally, the sequencing libraries were eluted in 25 μL DNA Purification Elution Buffer.

### Immunofluorescence

To confirm the number and type of cells in the biopsies, the biopsied and remaining parts of the blastocysts were subjected to CDX2 immunolabeling according to the method of Wydooghe et al. [31] with some modifications. Briefly, the biopsied and remaining parts were fixed in 10% (v/v) formalin neutral buffer solution (Fujifilm Wako Pure Chemical Corp., Osaka, Japan) for 1 h, washed in PBS containing 0.05% (v/v) Tween 20 (PBST) for 1 h and subsequently permeabilized with 0.5% (v/v) Triton X-100 in PBS for 1 h at room temperature. After washing in PBST for 1 h at room temperature, the samples were treated with blocking solution (PBST supplemented with 1% [w/v] BSA) for 1 h at room temperature and subsequently incubated in a ready-to-use primary anti-CDX2 antibody solution (BioGenex, Fremont, CA) overnight at 4 °C. After washing with blocking solution for 1 h at room temperature, the samples were incubated for 3 h at room temperature in the presence of Alexa Fluor 546-conjugated goat anti-mouse IgG (1:1000; Life Technologies, Carlsbad, CA). Nuclei were counterstained with 10 μg/mL Hoechst 33342 in PBST for 20 min. The samples were washed with PBST, mounted on slides with a droplet of VECTASHIELD mounting medium (Vector Laboratories, Burlingame, CA) and flattened with a coverslip. The slides were examined under a fluorescence microscope. The total number of cells was counted based on the Hoechst image, and the number of trophectoderm (TE) cells, which had been stained by both the anti-CDX2 antibody and Hoechst, was determined based on the merged images. The number of inner cell mass (ICM) cells was calculated by subtracting the number of TE cells from the total number of cells.

### WOW-CAT-qPCR

Quantitative PCR (qPCR) was conducted to assess the enrichment of specific histone modifications in the WOW-CAT libraries. The qPCR mixture (total volume: 10 μL) was prepared as follows: 5 μL THUNDERBIRD Next SYBR qPCR (Toyobo, Osaka, Japan), 1 μL library DNA, and 0.3 μL (10 μM) forward/reverse primers. PCR was performed with a StepOnePlus Real-time PCR system (Life Technologies) using the following program: 95 °C for 30 s, followed by 40 cycles at 95 °C for 5 s and 60 °C for 10 s with melt curve drawing. The primers used are listed in Table 1.

**Table 1:** Primers used for WOW-CAT-qPCR for sex identification

Gene	Primer sequence (5′–3′)	Product size (bp)
*XIST*	F: GGGTGGTAGAATCGGTCACA	71
	R: GGTAGCGAGGTGCTATGCTA	
*DDX3Y*	F: GAAAGGCGCGAACTCTGTCT	94
	R: TTCCGGTAGACCAACCTGTG	
*MUS81*	F: TCCAAAAGGCTGGTCCTGTC	70
	R: GGTTGGTACCGATCGCTGTA	

### Sex identification

We examined specific histone modifications that could be used for sex determination of the embryos. We focused on H3K4me3 modifications of the *XIST* and *DDX3Y* genes as putative female- and male-specific markers, respectively, from the WOW-CAT-next-generation sequencing (NGS) results. We conducted two experiments as follows. First, we biopsied the blastocysts as described above, and the biopsies were subjected to WOW-CAT-qPCR for *XIST* and *DDX3Y*. *MUS81* was used as an internal control. The remaining parts paired with the biopsies were subjected to conventional PCR-based sex identification using Y chromosome-specific repeat sequences as previously described [25, 32]. Then, we assessed the consistency of both methods. Second, to further examine the reliability of H3K4me3 modifications at *XIST* and *DDX3Y* as female- and male-specific markers, respectively, IVF with Y chromosome-sorted sperm was conducted, and the derived blastocysts were subjected to WOW-CAT-qPCR to detect H3K4me3 at *XIST* and *DDX3Y.*

### DNA sequencing and data processing

Sequencing and data processing were performed as previously reported [19]. Briefly, paired-end 150-base pair sequencing reads generated by a HiSeqX (Illumina) were quality checked, merged, and aligned to the bovine genome (Bos_taurus_UMD_3.1.1/bosTau8, June 2014) using Bowtie 2 [33]. Handling of sam and bam files was performed by Samtools (http://www.htslib.org/). Mapping duplicates were removed by Picard (http://broadinstitute.github.io/picard/). The generated bam files were converted to bigWig files by the bamCoverage tool of deepTools (https://deeptools.readthedocs.io/en/develop/) with counts-per-million normalization. The correlation plots between the experiments were made from bigWig files fed to deepTools. Average H3K4me3 signal profiles were generated by ngs.plot [34]. Peaks were visualized using Integrative Genomics Viewer [35]. The H3K4me3 peaks were called using MACS1.4 [36] as previously described [19]. The false positive rate (FPR) was calculated by the number of NTU-CAT or WOW-CAT peaks that did not overlap with ChIP-seq (Blastocysts 1 in our previous report [22]) but did overlap with the ATAC-seq peaks [37], divided by the total number of peaks [24].

### Publicly available data

For the comparison with the present WOW-CAT results, we used ChIP-seq (rep1 and rep3 of GSE16122) [22], NTU-CAT (Rep1–4 of https://zenodo.org/record/6002122) [19], and ATAC-seq (ICM_rep1 of GSE143658) [37] data, respectively.

### Supplementary Information

**Additional file 1.**

**Additional file 2.**

## Data Availability

The raw data generated in this study cannot be shared publicly from the viewpoint of genetic resource protection at this time point. The raw and processed data may be shared upon reasonable request to the corresponding author.
