# Supplementary material for: Evaluating histone modification analysis of individual preimplantation embryos
Source: BMC Genomics. 2024 Jan 18;25:75. doi: 10.1186/s12864-024-09984-8 (PMC10795292; doi:10.1186/s12864-024-09984-8)
Supplement: Supplementary file 1 — Additional file 1. [file 12864_2024_9984_MOESM1_ESM.pdf]

Supplementary Figure 1

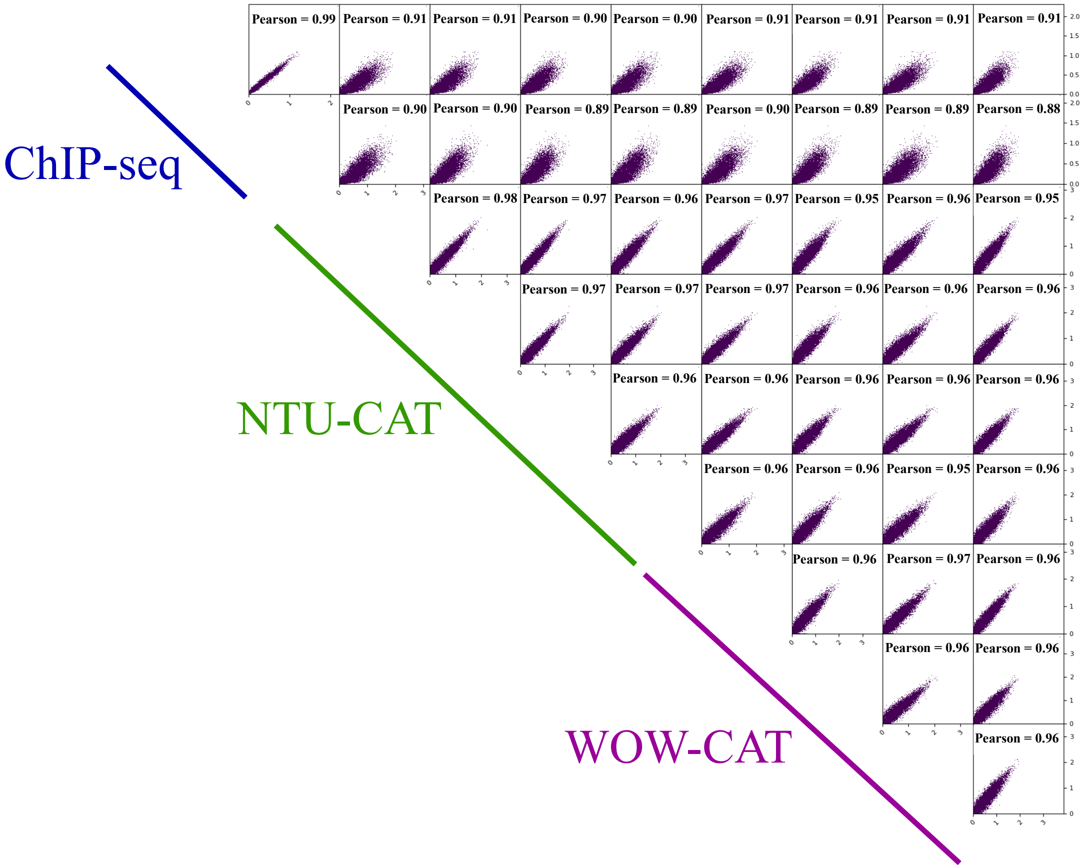

Supplementary Figure 1. Pairwise comparisons of H3K4me3 signals among the different methods and replicates. Scatterplots of these pairwise comparisons are shown with Pearson correlation coefficients. Bin sizes of 10 kb and natural log transformation after adding 1 were used for drawing with deepTools (https://deeptools.readthedocs.io/en/develop/).

Supplementary Figure 2

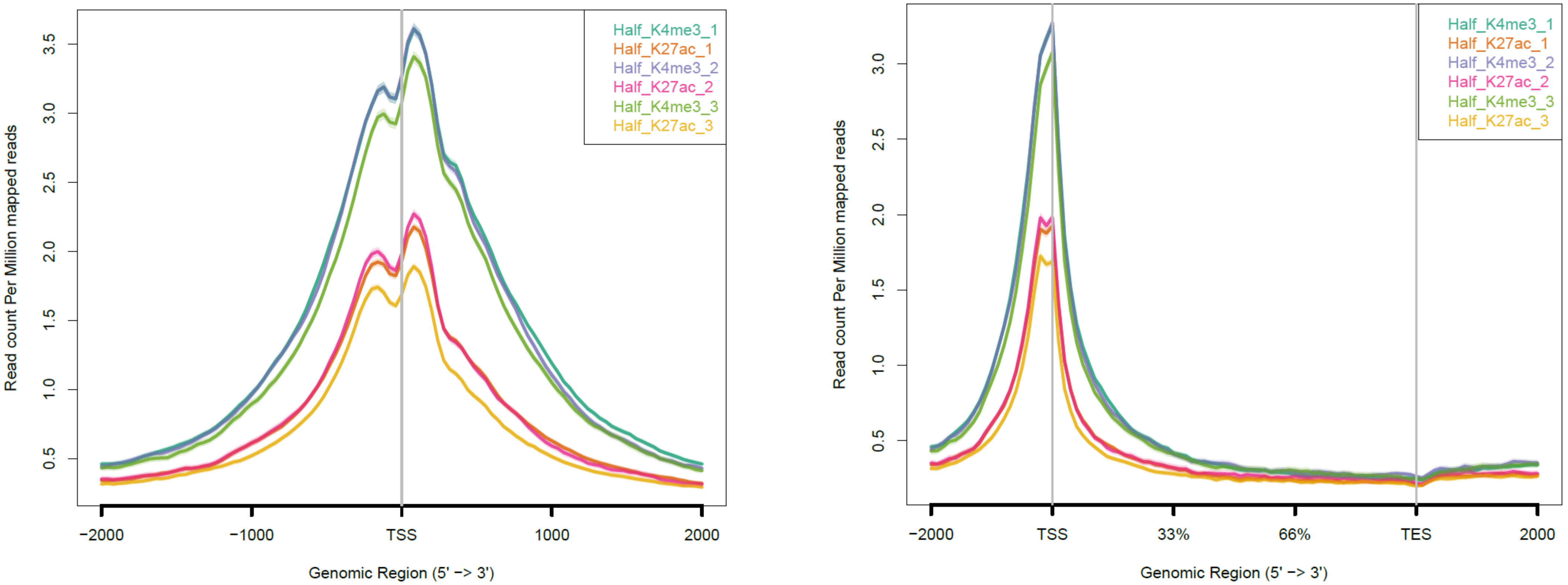

Supplementary Figure 2. Average profile plots of H3K4me3/H3K27ac signals around TSSs (left) and gene body (right) regions.

Supplementary Figure 3

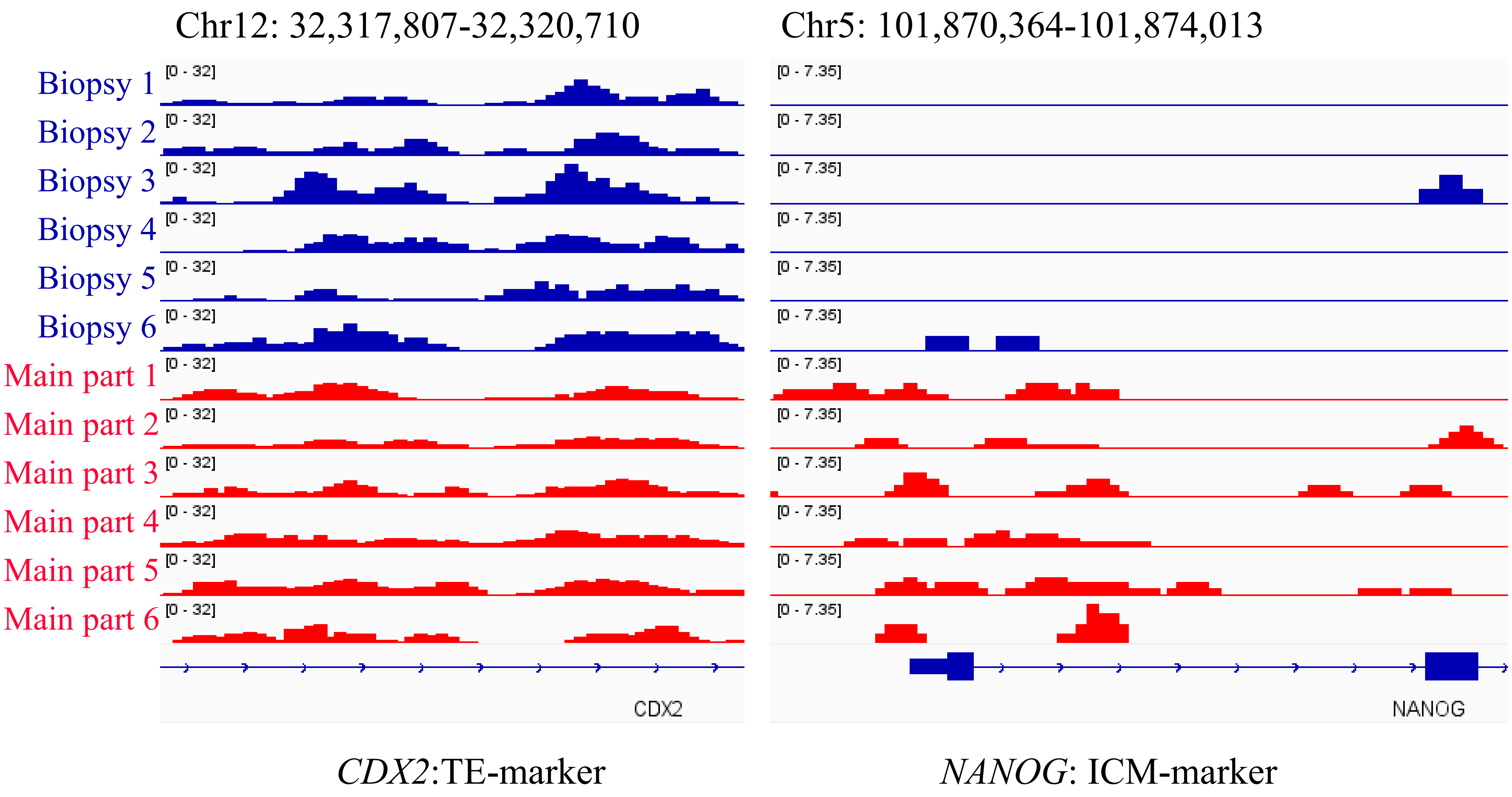

Supplementary Figure 3. The absence of H3K4me3 modifications at NANOG (an ICM-specific marker) in the biopsied parts.

Supplementary Figure 4

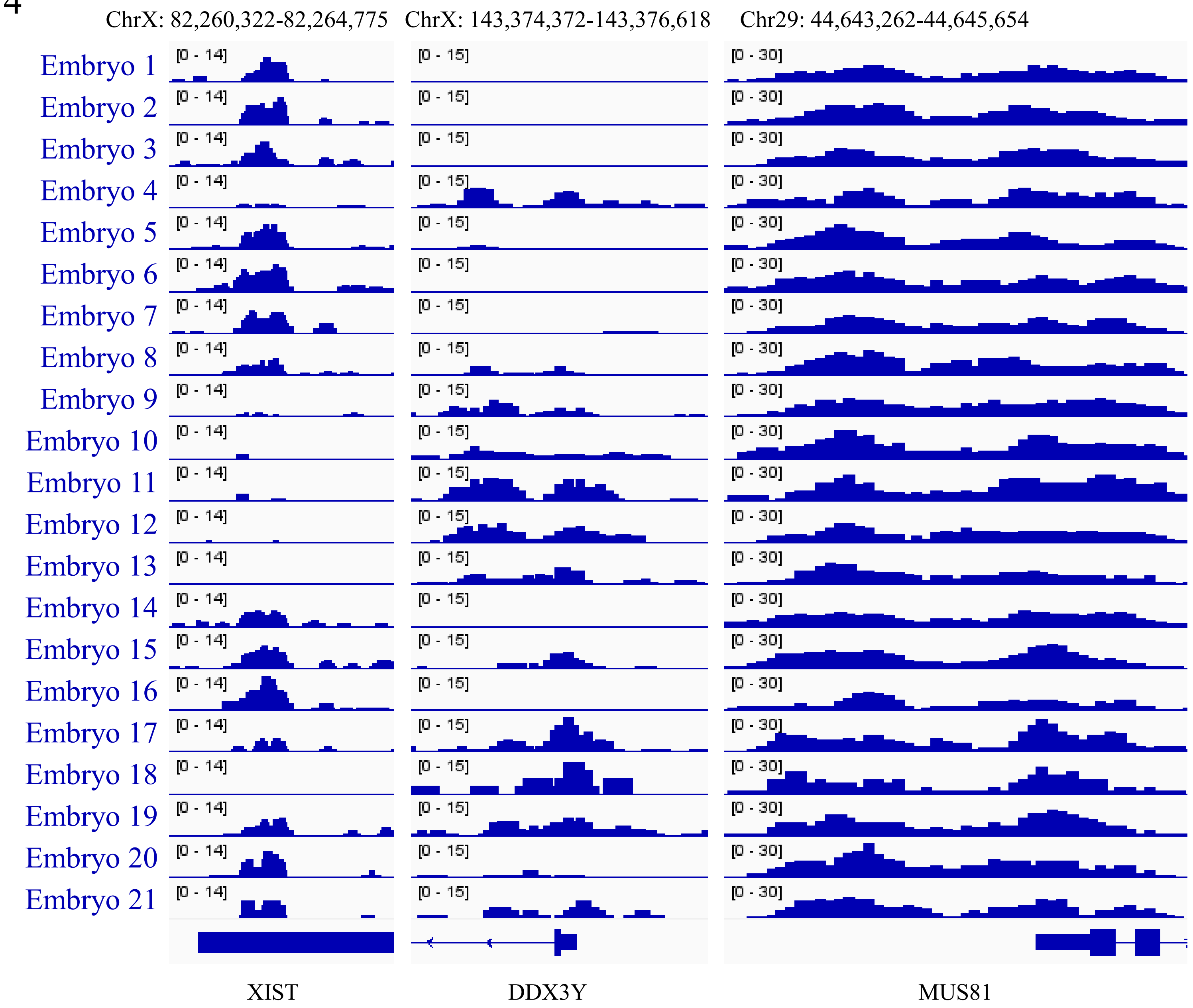

Supplementary Figure 4. NTU-CAT results at XIST and DDX3Y in multiple blastocysts.
