# Supplementary material for: Evaluating histone modification analysis of individual preimplantation embryos
Source: BMC Genomics. 2024 Jan 18;25:75. doi: 10.1186/s12864-024-09984-8 (PMC10795292; doi:10.1186/s12864-024-09984-8)
Supplement: Supplementary file 2 — Additional file 2. [file 12864_2024_9984_MOESM2_ESM.docx]

**Supplementary Table 1.** Comparison between NTU-CAT and WOW-CAT.

|  |  | NTU-CAT (13 samples) | WOW-CAT (13 samples) |
| --- | --- | --- | --- |
| Primary Antibody Binding | Reagent | Antibody buffer (975 µl) | Antibody buffer (425 µl) |
|  |  | Primary Antibody (13 µl) | Primary Antibody (1 µl) |
| Secondary Antibody Binding  (+ three washes) | Reagent | Secondary Antibody (13 µl)  Dig-wash buffer (8710 µl) | Secondary Antibody (1 µl)  Dig-wash buffer (400 µl) |
|  | Manipulation | 4 times well transfer for every 13 samples | 4 times liquid exchange in one well |
| pA-Tn5 Binding  (+ three washes) | Reagent | pA-Tn5 (13 µl)  Dig-300 buffer (8710 µl) | pA-Tn5 (1 µl)  Dig-300 buffer (400 µl) |
|  | Manipulation | 4 times well transfer for every 13 samples | 4 times liquid exchange in one well |
